# Supplementary material for: Empirical methods for controlling false positives and estimating confidence in ChIP-Seq peaks
Source: BMC Bioinformatics. 2008 Dec 5;9:523. doi: 10.1186/1471-2105-9-523 (PMC2628906; doi:10.1186/1471-2105-9-523)
Supplement: Additional File 1 — A variety of html documents from the USeq web site detailing the available applications, their best usage, output file type descriptions, command line menus, etc. [file 1471-2105-9-523-S1.zip › USeqUserGuides/JavaDoc.zip!/JavaDoc/trans/tpmap/TPMapLine.html]

TPMapLine


|  |  |  |
| --- | --- | --- |
| **Overview** \| **Package** \| **Class** \| **Use** \| **Tree** \| **Deprecated** \| **Index** \| **Help** | |  |
| **PREV CLASS**   **NEXT CLASS** | **FRAMES**    **NO FRAMES**     **All Classes** |  |
| SUMMARY: NESTED \| FIELD \| CONSTR \| METHOD | DETAIL: FIELD \| CONSTR \| METHOD |  |


---


## trans.tpmap Class TPMapLine

```
java.lang.Object
  trans.tpmap.TPMapLine
```

**All Implemented Interfaces:**: java.lang.Comparable

---

``` public class TPMapLine extends java.lang.Object implements java.lang.Comparable ```

Minimal tpmap line, chrom, start, nothing else.

---

**Constructor Summary**

`TPMapLine(java.lang.String line)`

`TPMapLine(java.lang.String[] items)`
  
          For parsing from an Agilent data file.

`TPMapLine(java.lang.String[] items,
boolean trueFalse)`
  
          For parsing from an Nimblegen NDF data file.

`TPMapLine(java.lang.String chr,
int pos,
int row,
int column,
java.lang.String sequence)`
  
          Coordinate are zero based!

`TPMapLine(java.lang.String chr,
int pos,
int row,
int column,
java.lang.String sequence,
boolean hybridizesForwardStrand)`
  
          Coordinate are zero based!

`TPMapLine(java.lang.String chrNum,
java.lang.String row,
java.lang.String column,
java.lang.String sequence)`
  
          For parsing from an Agilent promoter array data file.


| **Method Summary** | |
| --- | --- |
| `int` | `compareTo(java.lang.Object obj)` |
| `java.lang.String` | `getChromosome()` |
| `java.lang.String` | `getLine()` |
| `int` | `getPmX()` |
| `int` | `getPmY()` |
| `java.lang.String` | `getSequence()` |
| `int` | `getStart()` |
| `static boolean` | `saveTPMap(TPMapLine[] sortedTPMap, java.io.File tpmapFile)` |
| `void` | `setSequence(java.lang.String sequence)` |

**Methods inherited from class java.lang.Object**

`equals, getClass, hashCode, notify, notifyAll, toString, wait, wait, wait`

**Constructor Detail**

### TPMapLine

```
public TPMapLine(java.lang.String[] items)
```

:   For parsing from an Agilent data file.

---


### TPMapLine

```
public TPMapLine(java.lang.String chrNum,
                 java.lang.String row,
                 java.lang.String column,
                 java.lang.String sequence)
```

:   For parsing from an Agilent promoter array data file. Note there isn't a sequence for the oligo!

---


### TPMapLine

```
public TPMapLine(java.lang.String chr,
                 int pos,
                 int row,
                 int column,
                 java.lang.String sequence)
```

:   Coordinate are zero based!

---


### TPMapLine

```
public TPMapLine(java.lang.String chr,
                 int pos,
                 int row,
                 int column,
                 java.lang.String sequence,
                 boolean hybridizesForwardStrand)
```

:   Coordinate are zero based!

---


### TPMapLine

```
public TPMapLine(java.lang.String[] items,
                 boolean trueFalse)
```

:   For parsing from an Nimblegen NDF data file.

---


### TPMapLine

```
public TPMapLine(java.lang.String line)
```


**Method Detail**

### compareTo

```
public int compareTo(java.lang.Object obj)
```

:   **Specified by:**: `compareTo` in interface `java.lang.Comparable`

---


### saveTPMap

```
public static boolean saveTPMap(TPMapLine[] sortedTPMap,
                                java.io.File tpmapFile)
```

---


### getLine

```
public java.lang.String getLine()
```

---


### getChromosome

```
public java.lang.String getChromosome()
```

---


### getPmX

```
public int getPmX()
```

---


### getPmY

```
public int getPmY()
```

---


### getStart

```
public int getStart()
```

---


### getSequence

```
public java.lang.String getSequence()
```

---


### setSequence

```
public void setSequence(java.lang.String sequence)
```


---


|  |  |  |
| --- | --- | --- |
| **Overview** \| **Package** \| **Class** \| **Use** \| **Tree** \| **Deprecated** \| **Index** \| **Help** | |  |
| **PREV CLASS**   **NEXT CLASS** | **FRAMES**    **NO FRAMES**     **All Classes** |  |
| SUMMARY: NESTED \| FIELD \| CONSTR \| METHOD | DETAIL: FIELD \| CONSTR \| METHOD |  |


---
